# A Novel ESAT-6 Secretion System-Secreted Protein EsxX of Community-Associated *Staphylococcus aureus* Lineage ST398 Contributes to Immune Evasion and Virulence

**DOI:** 10.3389/fmicb.2017.00819

**Published:** 2017-05-05

**Authors:** Yingxin Dai, Yanan Wang, Qian Liu, Qianqian Gao, Huiying Lu, Hongwei Meng, Juanxiu Qin, Mo Hu, Min Li

**Affiliations:** ^1^Department of Laboratory Medicine, Renji Hospital, School of Medicine, Shanghai Jiao Tong UniversityShanghai, China; ^2^Institute of Analytical Chemistry and Synthetic and Functional Biomolecules Center, College of Chemistry and Molecular Engineering, Peking UniversityBeijing, China

**Keywords:** community-associated *Staphylococcus aureus*, ESAT-6 secretion system, secreted protein, EsxX, immune evasion, virulence

## Abstract

The ESAT-6 secretion system (ESS) has been reported to contribute to the virulence and pathogenicity of several *Staphylococcus aureus* strains such as USA300 and Newman. However, the role of the ESS in community-associated *S. aureus* (CA-SA) lineage ST398 in China is not well understood. By comparing the *ess* locus of ST398 with the published *S. aureus* sequence in the NCBI database, we found one gene in the *ess* locus encoding a novel WXG superfamily protein that is highly conserved only in ST398. LC-MS/MS and Western blot analysis revealed that this protein is a novel secreted protein controlled by the ST398 ESS, and we named the protein EsxX. Although EsxX was not under the control of the accessory gene regulator like many other virulence factors and had no influence on several phenotypes of ST398, such as growth, hemolysis, and biofilm formation, it showed important impacts on immune evasion and virulence in ST398. An *esxX* deletion mutant led to significantly reduced resistance to neutrophil killing and decreased virulence in murine skin and blood infection models, indicating its essential contribution to the evasion of innate host defense and virulence to support the pathogenesis of ST398 infections. The function of this novel secreted protein EsxX might help us better understand the role of the ESS in the virulence and epidemic success of the CA-SA lineage ST398.

## Introduction

*Staphylococcus aureus* is an important pathogen that causes a broad range of infections, including the majority of skin and soft tissue infections (SSTIs) and some life-threatening infections such as necrotizing pneumonia and fatal endocarditis (Lowy, 1998; Schijffelen et al., 2010). During infection, *S. aureus* expresses a wide array of secreted virulence factors to invade hosts and evade immune responses (Foster, 2009; Edwards and Massey, 2011; Spaan et al., 2013). Those virulence factors require secretion systems to translocate across the membrane.

A specialized ESAT-6 secretion system (ESS), similar to the type VII secretion system (T7SS) described in *Mycobacterium tuberculosis*, has been discovered in *S. aureus* (Burts et al., 2005). There are two homologous secretion factors of the *M. tuberculosis* T7SS effectors ESAT-6 and CFP-10, in the ESS of *S. aureus*, named EsxA and EsxB. It is reported that *esxA* and *esxB* deletion mutants resulted in decreased murine abscess formation by *S. aureus* (Burts et al., 2005). Other ESS substrates were reported subsequently, such as the proteins EsxC and EsxD. EsxC was shown to contribute to persistent infections (Burts et al., 2008), and EsxD was involved in the secretion of substrates (Anderson et al., 2013). A study indicated that EsxA and EsxB are involved in the modulation of apoptosis and release of ingested *S. aureus* from epithelial cells (Korea et al., 2014), focusing on the mechanistic function of secreted ESS substrates in pathogenesis. In addition, recent studies have revealed three new ESS components, EssD, EssE, and EssI, which contribute to the host immune response during infections (Anderson et al., 2017; Ohr et al., 2017). The majority of studies regarding ESS function were performed in USA300 and Newman (Burts et al., 2005, 2008; Anderson et al., 2011, 2013; Kneuper et al., 2014), two epidemic strains of community-associated *S. aureus* (CA-SA) in America. A few other strains were described, including RN6390, COL, SA113 (Kneuper et al., 2014), which belong to the same multi-locus sequence type (MLST) clonal complex (CC) as USA300 and Newman (CC8). However, there are strain-dependent differences in the *ess* locus among a broad range of *S. aureus* strains of different CC or sequence types, showing unexpected genetic diversity, which indicates that there might be strain-specific substrates and functions (Warne et al., 2016).

*Staphylococcus aureus* lineage ST398 was previously considered to be livestock-associated (LA) and has been reported in European countries and North America (Voss et al., 2005; Witte et al., 2007; Smith et al., 2009; Smith and Pearson, 2011; Wassenberg et al., 2011). However, the emergence of ST398 methicillin-sensitive *S. aureus* (MSSA) in community households in northern Manhattan has been observed (Bhat et al., 2009), independent of animal contact. Additionally, there has been a fatal infection caused by ST398 reported in Japan, and the isolate is not likely to be associated with livestock (Koyama et al., 2015). Additionally, an epidemiological study in China indicates that lineage ST398 could be CA-SA rather than LA-SA (Chao et al., 2014). The extraordinary virulence of CA-SA allows these strains to infect healthy individuals and spread easily from person to person. Therefore, the emerging CA-SA lineage ST398 requires more consideration. However, the molecular underpinnings of the virulence characteristics and epidemiological success of this CA-SA lineage remain poorly understood.

Recently, one of our studies showed that the prevalence of the CA-SA ST398 is increasing in China, and we have reported the contribution of the ESS to the virulence of the emerging CA-SA lineage for the first time (Wang et al., 2016). Compared with USA300 and Newman, our knowledge regarding the structure and function of the ESS of ST398 is still limited. The role of the ESS in ST398 needs further investigation, which would help us better understand the virulence and epidemiological success of this CA-SA lineage.

In the present study, we compared the *ess* locus between ST398 with the published *S. aureus* sequence in the NCBI database and found one gene that is highly conserved in *S. aureus* lineage ST398. This gene is located downstream of *esxB*, and its encoded protein has a unique LXG domain belonging to the WXG superfamily. We determined that the secretion of this protein is controlled by ESS, identified its subcellular localization, and named the protein EsxX. The role of EsxX has been studied by comparing ST398 wild type and an isogenic deletion mutant using *in vitro* and *in vivo* experiments. According to our results, this novel secreted protein EsxX did not impact the secretion of EsxA or EsxB. It was not under the control of accessory gene regulator (*agr*) and did not influence the growth, hemolysis, or biofilm formation of ST398. However, we demonstrated that EsxX promoted neutrophil lysis and contributed to the evasion of elimination by human neutrophils, indicating that the protein has the potential to interfere with innate defense. Furthermore, in experimental skin and blood infections, EsxX showed a significant contribution to the virulence and pathogenicity of ST398.

## Materials and Methods

### Ethics Statement

All animal experiments were carried out in accordance with the Guide for the Care and Use of Laboratory Animals of the Chinese Association for Laboratory Animal Sciences (CALAS) and the protocol was approved by the ethics committee of Renji Hospital, School of Medicine, Shanghai Jiao Tong University, Shanghai, China. Human heparinized venous blood was obtained from healthy individuals in accordance with a protocol approved by the ethics committee of Renji Hospital, School of Medicine, Shanghai Jiao Tong University, Shanghai, China. All individuals gave written informed consent in accordance with the Declaration of Helsinki prior to donating blood.

### Bacterial Strains, Plasmids, Oligonucleotides, and Growth Conditions

Clinical *S. aureus* isolates were obtained from a comprehensive teaching hospital in Shanghai, China (Renji Hospital, School of Medicine, Shanghai Jiao Tong University). Bacteria were identified as staphylococci by classic microbiological methods, including Gram staining, catalase and coagulase activity on rabbit plasma. *S. aureus* strains were further categorized by VITEK2 automated systems (BioMérieux, France). CA-SA was defined as previously described (Li et al., 2016). Isolates were obtained either from an outpatient or from an inpatient <24 h after hospital admission and lacked the following risk factors: contact with the hospital environment in the preceding 6 months, residence in a long-term care facility in the preceding 12 months, *S. aureus* infection in the preceding 12 months, and presence of a central vascular catheter at the time of infection. All bacterial strains and plasmids used in this study are listed in **Table 1**. *S. aureus* was grown in tryptic soy broth (TSB; Oxoid) with 0.25% glucose or on agar plates at 37°C, and *Escherichia coli* was routinely grown in Luria-Bertani medium (LB; Oxoid). When necessary, antibiotics were used at the following concentrations: ampicillin, 100 μg/ml; chloramphenicol, 10 μg/ml. All oligonucleotides used in this study are listed in **Table 2**.

**Table 1:** Bacterial strains and plasmids used in this study.

Strains/plasmids	Relevant genotype and property	Source
***S. aureus***		
RN4220	derived from NCTC8325-4;r-m+	de Azavedo et al., 1985
RJ-ST398	CA-SA clinical isolate	Wang et al., 2016
RJ-ST398Δ *essB*	RJ-ST398 *essB* mutant	Wang et al., 2016
RJ-ST398Δ*esxX*	RJ-ST398 *esxX* mutant	This study
RJ-ST398Δ*esxX*(pOS1*esxX*)	RJ-ST398 *esxX* mutant with pOS1- *esxX*	This study
RJ-ST398Δ*esxX*(pOS1)	RJ-ST398 *esxX* mutant with pOS1	This study
RJ-ST398Δ *agr*	RJ-ST398 *agr* mutant	Wang et al., 2016
***E. coli***		
DH5α	*endA*1 *recA*1 *gyrA*96 *thi-1 hsdR*17(rK-mK+) *relA*1 *supE*44 (*lacZYA-argF*)U169 F-80d*lac*ZM15 *deoR phoA*	Invitrogen
BL21	*E. coli B F- dcm ompT hsdS*(rB- mB-) gal For recombinant protein production	Amersham Biosciences
***Plasmids***		
pKOR1	cmR and ampR, temperature-sensitive vector for allelic replacement via lambda recombination and *ccdB* selection	Bae and Schneewind, 2006
pKOR1Δ*esxX*	Vector for allelic replacement of *esxX* in *S. aureus*	This study
pOS1	*E. coli/Staphylococcus* shuttle cloning plasmid, cmR, ampR	Bubeck Wardenburg et al., 2006
pOS1*esxX*	pOS1 with insertion of *esxX* gene	This study


**Table 2:** Oligonucleotides used in this study.

Oligonucleotide	Sequence
***Oligonucleotides for isogenic deletion mutants***	
esxX-att1	ggggacaagtttgtacaaaaaagcaggctgatggtatgagtaaacttaagga
esxX-rev1	tttatactcctttactcttttat
esxX-rev2	aagagtaaaggagtataaaaaggagatttaaaatgaataat
esxX-att2	gggg accactttgtacaagaaagctgggtggtaaaagggcaattgaagg
***Oligonucleotides for genetic complementation***	
EsxX- Sma1-F	gagcccgggatggggaataaaataaaaatgtcag
EsxX-BamH1-flag-R	gagggatccttacttatcgtcgtcatccttgtaatcaaatacattgcttaacgtttt
***Oligonucleotides for qRT- PCR***	
gyrB-F	caaatgatcacagcatttggtacag
gyrB-R	cggcatcagtcataatgacgat
esxX-F	caactggtacggcaatcggt
esxX-R	tgacttgccaccaacaatctt
***Oligonucleotides for construction and purification of His-tagged fusion protein***	
EsxX-EcoR1-F	gaggaattcatggggaataaaataaaaatgtcag
EsxX-BamH1-R	Gagggatccttaaaatacattgcttaacgtttt


### Molecular Typing

Multi-locus sequence typing (MLST) was performed as previously described (Enright et al., 2000). PCR amplicons of seven *S. aureus* housekeeping genes (*arcC*, *aroE*, *glpF*, *gmk*, *pta*, *tpi*, *and yqiL*) were obtained from chromosomal DNA. The sequences of the PCR products were compared with the existing sequences available at the MLST website^1^.

### Sequence Alignment and Conservation Analysis

Sequence alignment of *esxX* gene among clinical *S. aureus* ST398 isolates was performed using MEGA software. The *esxX* gene sequence was used for BLAST in the NCBI database. The amino acid sequence alignment of EsxX both from clinical isolates and the NCBI database were analyzed by GENEDOC software.

### Mass Spectrometric Analysis

Bacteria were grown to an OD_600_ of 5.0, and filtrates of the cultures were precipitated with 10% trichloroacetic acid (TCA and washed 3 times with ice-cold acetone. The samples were then resuspended in sample buffer (50 mM Tris-HCl pH 8.0, 4% SDS) and then mixed with protein loading buffer and boiled for 10 min. A 12% SDS-PAGE was used to pre-fractionate the secreted proteins and then the protein samples were subjected to in-gel digestion. Finally, the extracted peptides were vacuum-dried prior to LC-MS/MS analysis on a nanoflow liquid chromatography instrument (EASY-nLC 1000, Thermo Scientific) coupled to an ion trap mass spectrometer (LTQ Velos Pro, Thermo Scientific) in the data-dependent mode. The detailed LC-MS/MS settings for this procedure have been described previously (Hu et al., 2014).

### Allelic Gene Replacement by Homologous Recombination and Genetic Complementation

For gene deletion in CA-SA ST398, a representative clinical isolate (RJ-ST398) was chosen; this isolate was recovered from an abscess of a patient with a skin infection (Wang et al., 2016). The homologous recombination procedure using the plasmid pKOR1 was performed as described (Bae and Schneewind, 2006). DNA fragments for the upstream and downstream sequences of *esxX* were PCR amplified from the chromosomal DNA of RJ-ST398, and overlap PCR was used to obtain a fused PCR product. This product was cloned into pKOR1 using a Clonase reaction and *attB* sites, yielding the plasmid pKOR1Δ*esxX*. The plasmid pKOR1Δ*esxX* was transferred via electroporation into *S. aureus* RN4220 and then into RJ-ST398. The allelic replacement procedure was performed as described by others (Vuong et al., 2003). Proper integration was verified by PCR amplification from genomic DNA using *esxX*-att1 and *esxX*-att2 primers and sequencing of the PCR product. For genetic complementation, the *esxX* gene was amplified by PCR with the primers *esxX*-SmaI-F and *esxX*-BamHI-flag-R. The *esxX* complementation plasmid was generated by cloning the *esxX* gene into vector pOS1 and was then transferred into RJ-ST398. The plasmid control strain was obtained by transferring the pOS1 plasmid into RJ-ST398.

### Staphylococcal Fractionation

Overnight cultures were diluted 1:100 into 10 ml of TSB and incubated at 37°C with shaking at 200 rpm until they had grown to an OD_600_ of 2.0 (mid-exponential growth phase), and 1.5 ml of culture was centrifuged at 10,000 × *g* for 4 min to separate the cells from the supernatant. Proteins in 1 ml of supernatant were precipitated with 10% TCA using 3 ice-cold acetone washes and then resuspended in sample buffer (50 mM Tris-HCl pH 8.0, 4% SDS). The cells were resuspended in 80 μl of TE buffer (10 mM Tris-HCl, 1mM EDTA, pH 8.0). Lysostaphin (100 μg/ml) was added for a 10 μg/ml final concentration, and the samples were incubated for 30 min at 37°C. Supernatant (S) and cell lysate (CL) samples were both mixed with protein loading buffer and boiled for 10 min. For subcellular localization, 2 ml cultures were centrifuged as described above, and the supernatants were precipitated with TCA. The cell pellet of a 2 ml culture were was with TSM buffer, and then suspended in 500 μl of TSM buffer containing 10 μl of lysostaphin (2 mg/ml stock) and incubated at 37°C for 30 min. The protoplasts were collected by centrifugation at 10,000 × *g* for 10 min, and the cell wall fraction (W) was precipitated with TCA. Protoplasts were suspended in 5ml of membrane buffer (0.1 M Tris-HCl, 0.1 M NaCl, pH 7.5, 0.01 M MgCl_2_) and sonicated on ice at full power for 10 s. Soluble proteins (C, cytoplasmic fraction) were separated from insoluble materials and membranes (M, membrane fraction) by ultracentrifugation at 45k rpm for 30 min, at 4°C. All samples were precipitated with TCA before Western blotting.

### Western Blot Analysis

Equal volumes (10 μl) of all proteins of interest were separated by SDS-PAGE and transferred onto nitrocellulose membranes (Invitrogen). After blocking, the membranes were incubated with specific antiserum at 4°C overnight and then incubated with a horseradish peroxidase-conjugated secondary antibody at room temperature for 1 h. Images of Western blots were acquired using a Tanon-5200 system. In total, eight specific antibodies were used for Western blot analysis in this study, including α-EsxX, α-EsxA, α-EsxB (generated by GLBiochem, China); α-SrtA, α-SeaS, and α-SeaR (kindly provided by Taeok Bae, Indiana University School of Medicine-Northwest, Gary, Indiana); α-Hla (Abcam) and α-Spa (Sigma-Aldrich).

### Quantitative Reverse-Transcription (RT) PCR

Complementary DNA (cDNA) was synthesized from total RNA using the QuantiTect RT system (Qiagen) according to the manufacturer’s instructions. Oligonucleotide primers were designed using Primer Express (**Table 2**). The resulting complementary DNA and negative control samples were amplified using the QuantiTect SYBR green PCR kit (Qiagen). Reactions were performed in a MicroAmp Optical 96-well reaction plate using a 7500 Sequence Detector (Applied Biosystems). Standard curves were determined for each gene using purified chromosomal DNA at concentrations of 0.005–50 ng/ml. All quantitative reverse-transcription polymerase chain reaction (qRT-PCR) experiments were performed in duplicate with *gyrB* as an internal control.

### Analysis of Hemolytic Capacity

Strains were grown to the mid-exponential growth phase (4 h), and equal amounts (2 μl) of cells were spotted on sheep blood agar plates. After incubation at 37°C for 24 h, the zones of hemolysis (diameter) were measured.

### Semiquantitative Biofilm Assay

Semiquantitative biofilm assays were performed as described previously (Vuong et al., 2003). Cells were fixed by Bouin’s fixative. The fixative was removed after 1 h incubation, and wells were washed with phosphate-buffered saline (PBS). Organisms in the wells were then stained with crystal violet, and the floating stain was washed off with slow-running water. After drying, the stained biofilm was scanned with a Micro ELISA autoreader (BioRad) at 570 nm.

### Neutrophil Lysis and Bacterial Survival Assays

Human neutrophils were isolated from heparinized venous blood of healthy individuals with a standard method. Bacteria were grown to the mid-exponential growth phase and incubated with neutrophils at a 10:1 ratio (MOI 10) at 37°C for 3h. PBS with 0.1% Triton-X100 (100 μl) was used to determine 100% lysis. Lysis was measured using a lactate dehydrogenase (LDH) cytotoxicity detection kit (Roche) according to the manufacturer’s protocol as described elsewhere (Li et al., 2012). To determine bacteria survival rates, 100 μl of the culture above was serially diluted and plated on TSA containing 5% sheep blood for *S. aureus* colony forming units (CFU) counting.

For microscopic evaluation of neutrophil killing after phagocytosis and intracellular *S. aureus* counting, overnight cultures were diluted 1:100 into 10 ml of TSB and incubated at 37°C with shaking at 200 rpm to the mid-logarithmic growth phase (4 h). The cells were washed and resuspended in sterile PBS at a concentration of 1 × 10^10^ CFU/ml. *S. aureus* cells were mixed with heparinized venous blood at a ratio of blood/bacterial solution of 10:1 (v/v) and incubated at 37°C. After the indicated times of incubation, blood smears were prepared and cells were stained with a modified Wright-Giemsa stain. The number of neutrophils lysed by *S. aureus* among the total number of neutrophils that had ingested *S. aureus* (*n* = 100 analyzed) was calculated by microscopic examination at 30 min after incubation. The number of intracellular *S. aureus* after 30 min of infection was counted using 20 neutrophils per group.

### Mouse Skin Abscess and Bacteremia Models

The skin abscess model was performed as described elsewhere (Wang et al., 2007). Outbred, immunocompetent hairless female mice between 4 and 6 weeks of age were used for the experiments. Anesthetized mice were inoculated subcutaneously on the back with 100 μl of PBS containing 10^7^ CFU of live *S. aureus* or an equal volume of PBS alone. The next day, abscess length (L) and width (W) values were measured to calculate the area of abscesses with the formula area (A) = π (L × W)/2. The abscess skin tissue was excised, washed with saline, and fixed in 4% formalin (Sigma-Aldrich). Paraffin embedding and hematoxylin and eosin (H&E) staining were performed.

For the bacteremia model, BALB/c female mice between 4 and 6 weeks of age were used. *S. aureus* strains were grown to mid-logarithmic phase, washed once with sterile PBS and then suspended in PBS. Each mouse of one group was injected with 100 μl of PBS containing 10^8^ CFU of live *S. aureus* into the retro-orbital vein, and another group was injected with 100 μl of PBS containing 10^9^ CFU of each for survival analysis. Control animals received sterile PBS only. After inoculation, the health of the mice and the advancement of their disease state were monitored every day. The mice were euthanized immediately if they showed signs of respiratory distress, mobility loss or inability to eat and drink. The surviving mice injected with 10^8^ CFU were euthanized 4 days after injection. Kidneys were excised, washed with saline, and one kidney was fixed in 4% formalin (Sigma-Aldrich). Paraffin embedding and hematoxylin and eosin (H&E) staining were performed. The other kidney was homogenized in 0.5 ml of PBS, and the homogenized kidney tissue was diluted and plated on TSB agar for the determination of CFU numbers. All surviving mice injected with 10^9^ CFU were euthanized at 5 days.

### Statistics

Statistical analysis was performed using Graph Pad Prism, version 6.01. The data were analyzed using unpaired *t*-tests to compare two different conditions. All error bars show the standard error of the mean (SEM).

## Results

### A Novel Protein EsxX with Secretion Potential Is Highly Conserved in ST398

**FIGURE 1:**
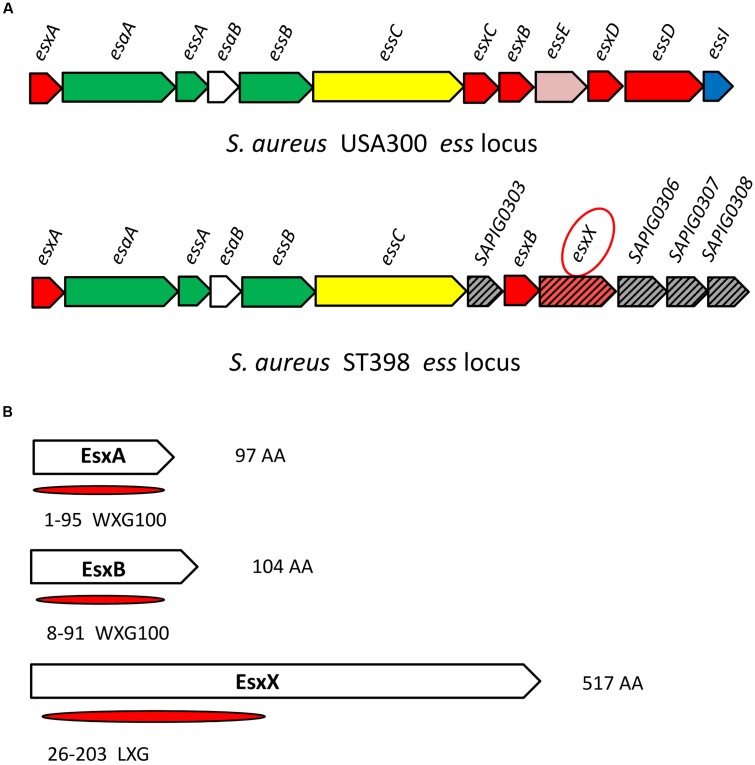
**Location of *esxX* in the CA-SA ST398 *ess* locus and its domain organization.** **(A)** *ess* locus of USA300 and ST398. Gene and protein colors indicate FtsK-SpoIIIE domain-ATPases (yellow); secreted protein (red); transmembrane protein (green); cytoplasmic protein (white); hypothetical protein (slash pattern); protein of unknown function (gray); EssE (pink); EssI (EssD inhibitor, blue). **(B)** EsxA and EsxB, both members of the WXG100 superfamily. EsxX, a 517 aa protein, has an LXG domain at the N-terminal and belongs to the WXG superfamily.

The *ess* locus of USA300 or Newman has been well characterized. There are six core genes from *esxA* to *essC* and several other genes of the system (**Figure 1A**), in which the EsaA, EssA, EssB, and EssC proteins are membrane-located components of the secretion machinery, EsaB is a regulator, and EsxA, EsxB, EsxC, and EsxD are exported effector proteins (Burts et al., 2005, 2008; Anderson et al., 2011; Kneuper et al., 2014); three additional components of the ESS pathway, EssE, EssD, and EssI, were recently identified (Anderson et al., 2017; Ohr et al., 2017). Compared with USA300 or Newman, the *ess* locus of ST398 contains the same six core genes from *esxA* to *essC*, as well as another exported effector encoded gene *esxB*, but the *esxC*, *esxD*, *essE*, *essD*, and *essI* genes are all absent; instead, there are other genes of unknown function (**Figure 1A**). We found one gene located downstream of the *esxB* gene (**Figure 1A**) that encodes a 517 amino acid protein we named EsxX. The ESS has two identified secreted virulence factors, EsxA and EsxB, both of which are members of the WXG100 superfamily. The WXG motif of EsxA and EsxB is referred to as a signature sequence of ESAT-6-like proteins that can be secreted by ESS (Pallen, 2002). EsxX, which also belongs to the WXG superfamily, has a unique LXG domain at the N-terminal (**Figure 1B**; Schneewind and Missiakas, 2012), indicating that the protein might have secretion potential. This is consistent with a study proposing that EsxX might be secreted by the ESS (Warne et al., 2016). Furthermore, we performed sequence alignment of *esxX* among clinical *S. aureus* isolates and strains in the NCBI database and found that it is highly conserved in *S. aureus* lineage ST398. 61 isolates of *S. aureus* ST398 derived from infected patients and 15 isolates derived from cattle suffering from mastitis were collected in this study. Whole genome sequencing of the 76 isolates was performed, and the present study used the sequence of the *esxX* gene of the 76 clinical isolates for sequence alignment. According to the alignment results, 86.8% of isolates (66/76) showed 100% sequence identity, and the other 13.2% of isolates showed 99% identity, differing only by one point mutation (Supplementary Table 1). In the NCBI BLAST database, there is no homologous protein of EsxX in USA300 or other *S. aureus* strains. Only one ST582 and four ST15 strains were discovered to have 96% sequence identity and they had lower scores and more gaps than the other strains belonging to ST398 which showed 100 or 99% sequence identity of *esxX* (Supplementary Table 2). These findings suggest that EsxX is a clone-specific protein of ST398 that might have special functions.

### Secretion of EsxX Is Controlled by the ESS of ST398 and it Has No Effect on the Secretion of Other ESS-Secreted Factors

**FIGURE 2:**
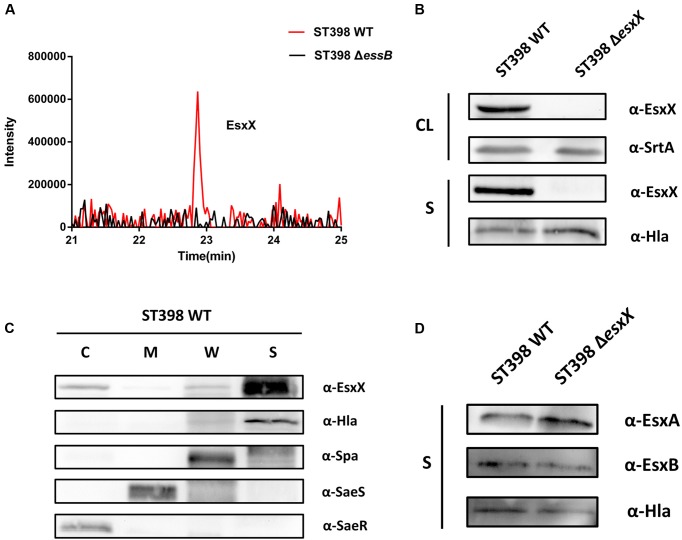
**EsxX is a secreted protein of the ESS of ST398 and it has no effect on the secretion of other ESS-secreted factors.** **(A)** Detection of secreted proteins from ST398 wild type and *essB* deletion strains by mass spectroscopy. Peaks of peptides derived from EsxX are present in wild type samples but absent in samples from the *essB* deletion mutant. **(B)** *S. aureus* ST398 cultures were separated into cell lysate (CL) and supernatant (S) fractions. Proteins were detected by Western blotting with specific antibodies (α-EsxX, α-SrtA, α-Hla). **(C)** *S. aureus* ST398 cultures were fractionated into the cytoplasmic (C), membrane (M), cell wall (CW), and supernatant (S) compartments. Proteins were detected by Western blotting with specific antibodies (α- EsxX, α-Hla, α-Spa, α-SeaS, α-SeaR). **(D)** Supernatant (S) fractions of ST398 wild type and *agr* mutant cultures were obtained. Proteins were detected by Western blotting with specific antibodies (α-EsxA, α-EsxB, α-Hla).

Our previous study confirmed that ST398 ESS is responsible for the secretion of EsxA and EsxB via LC-MS/MS analysis of ST398 wild type versus *essB* deletion mutant culture filtrates (Wang et al., 2016). It is worth mentioning that in the same LC-MS/MS analysis, EsxX was not detectable in the *essB* deletion mutant culture filtrate (**Figure 2A**), indicating that its secretion is controlled by the ESS of ST398. To verify the secretion property of EsxX, we constructed an isogenic gene deletion mutant of *esxX* by a representative ST398 isolate selected previously (Wang et al., 2016). We performed a Western blot to determine whether EsxX was secreted. EsxX was detected both in the CL and the supernatant of ST398 using specific rabbit antisera (**Figure 2B**). Sortase-A and Hla were used as controls for proteins in the CL and the supernatant, respectively. Furthermore, to examine the subcellular localization of EsxX, cultures of ST398 were separated into cytoplasmic (C), membrane (M), cell wall (W), and supernatant (S) fractions (**Figure 2C**). Proteins in all fractions were examined by Western blotting with specific antibodies. EsxX was found predominantly in the supernatant and partially in the cytoplasm. As a control, Hla was detected in the supernatant, whereas Spa resided in both the cell wall and supernatant fraction, SaeS in the membrane, and SaeR in the cytoplasm (**Figure 2C**). Together, these results demonstrate that EsxX is a novel secreted protein controlled by ST398 ESS. Because EsxA and EsxB are two ESS effectors that play important roles in the function of ESS, we then used Western blotting to analyze whether EsxX impacts their secretion. EsxA and EsxB were detected in the supernatant fraction of both ST398 wild type and *esxX* mutant bacteria at the same level (**Figure 2D**), which suggested that the ESS was still completely functional when the *esxX* gene was knockout.

### EsxX Is Not under the Control of *agr* and Does Not Affect the Growth, Hemolysis or Biofilm Formation of ST398

**FIGURE 3:**
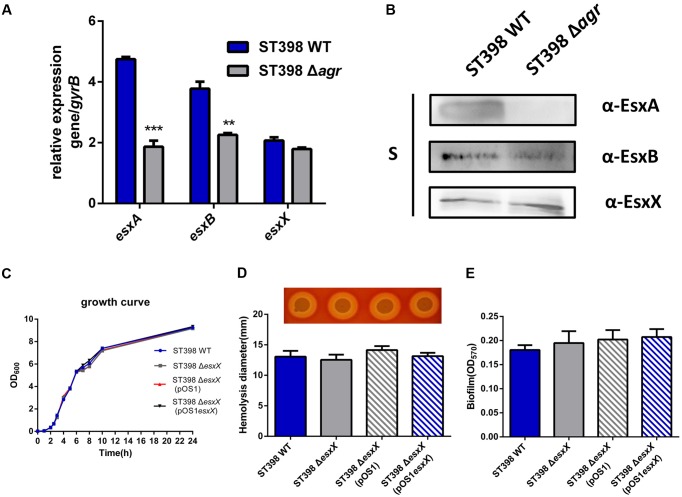
**EsxX is not under the control of *agr* and the role of EsxX in *S. aureus* ST398 phenotypes.** **(A)** Gene transcription of *esxA*, *esxB*, and *esxX* and **(B)** protein expression of EsxA, EsxB, and EsxX in ST398 wild type versus an *agr* deletion mutant as determined by qRT-PCR and Western blotting. ^∗∗^*p* < 0.01; ^∗∗∗^*p* < 0.001 (unpaired *t*-tests). **(C)** Growth of ST398 wild type versus the *esxX* mutant. **(D)** Zones of hemolysis (diameter) due to the wild type strain versus the *esxX* deletion mutant, the complemented mutant and the deletion mutant harboring a control plasmid. **(E)** Detection of biofilm formation in wild type strain versus the *esxX* deletion mutant, the complemented mutant and the deletion mutant harboring a control plasmid.

EsxA and EsxB, two canonical secreted proteins of ESS, have been described as secreted virulence factors in other *S. aureus* strains (Burts et al., 2005; Zhou et al., 2013). Previous work had shown that the *esxA* and *esxB* genes were under *agr* control at a transcriptional level (Wang et al., 2016). Because EsxX is a novel secreted protein of ST398 ESS, we questioned whether it had similar functions to the two Esx proteins. Quantitative RT-PCR and Western blotting were used to investigate EsxA, EsxB, and EsxX expression in ST398 wild type versus *agr* deletion mutant (**Figure 3A**). We found that EsxA and EsxB were *agr*-regulated, while EsxX was not (**Figure 3B**). We also found that there is no difference in growth between ST398 wild type and an *esxX* mutant (**Figure 3C**). To study the role of EsxX in ST398, we examined the capacity of the bacteria to induce hemolysis, one of the most important virulence phenotypes of *S. aureus*, but found no difference between the wild type and the *esxX* mutant strain (**Figure 3D**). *S. aureus* has been reported to form biofilms during infection, which promotes persistence and contributes to its successful colonization, making it difficult to eliminate the infection (Otto, 2008). However, the EsxX had no impact on biofilm formation (**Figure 3E**).

### EsxX Promotes Neutrophil Lysis and Contributes to Evasion of Innate Host Defense in ST398 Infections

**FIGURE 4:**
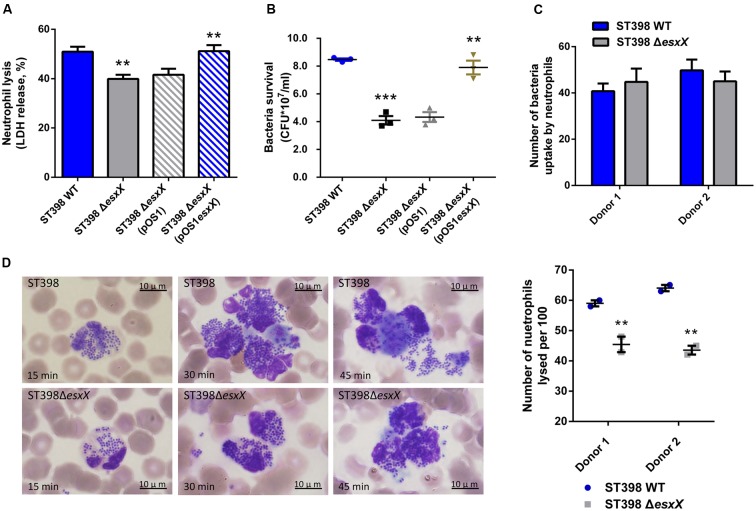
**EsxX promotes neutrophil lysis and contributes to evasion of elimination by human neutrophils.** **(A)** Neutrophil lysis. Human neutrophils were isolated from heparinized venous blood of healthy individuals. Bacteria were grown to the mid-logarithmic phase and incubated with neutrophils at a 10:1 ratio. Neutrophil lysis was determined by measuring the release of LDH. **(B)** Bacterial survival. Bacterial survival was measured by incubating a mixture of bacteria and neutrophils and plating for CFUs. ^∗∗^*p* < 0.01; ^∗∗∗^*p* < 0.001 (unpaired *t*-tests of the deletion mutant versus wild type strain and the complemented mutant versus the deletion mutant harboring a control plasmid). **(C,D)** Microscopic evaluation of neutrophil phagocytosis. Blood smears were obtained at different time points in the incubation of bacteria with neutrophils. Ingestion of bacteria and neutrophil lysis were analyzed by light microscopy using a modified Wright-Giemsa stain. **(C)** The numbers of intracellular *S. aureus*. Twenty neutrophils per group were used to examine the number of intracellular *S. aureus* after 30 min of infection. (*P* > 0.05; **D)** Neutrophil lysis after phagocytosis. The number of lysed neutrophils among 100 neutrophils that had ingested bacteria is shown on the right for two different donors. ^∗∗^*p* < 0.01 (unpaired *t*-tests).

Recently, EsxA was reported to play a role in the immune response to invasive *S. aureus* disease (Zhang et al., 2015) and to delay apoptosis and contribute to the release of *S. aureus* from epithelial cells, together with EsxB (Korea et al., 2014). These reports inspired us to investigate whether the novel secreted protein EsxX impacts the interaction between *S. aureus* and host cells. We selected neutrophils because neutrophils represent the first line of defense against a *S. aureus* infection. We analyzed the lysis of neutrophils and the degree of bacterial survival by incubating wild type and *esxX* mutant bacteria with human neutrophils. Compared with the wild type strain, the *esxX* mutant showed significantly decreased neutrophil lysis (**Figure 4A**). The low bacterial survival of the *esxX* mutant (**Figure 4B**) showed that the mutant was more likely to be eliminated than the wild type. Further, a microscopic analysis was performed. We examined the numbers of intracellular *S. aureus* after 30 min of infection, and the data showed no impact of EsxX on uptake by neutrophils (**Figure 4C**), while the count of lysed neutrophils after ingestion (**Figure 4D**) suggested that EsxX contributed to neutrophil lysis after phagocytosis. These results indicated that EsxX impacts the capacity of ST398 to interact with human neutrophils and thus plays an important role in the evasion of innate host defenses.

### EsxX Makes a Significant Contribution to Virulence of ST398 in Skin and Blood Infection

**FIGURE 5:**
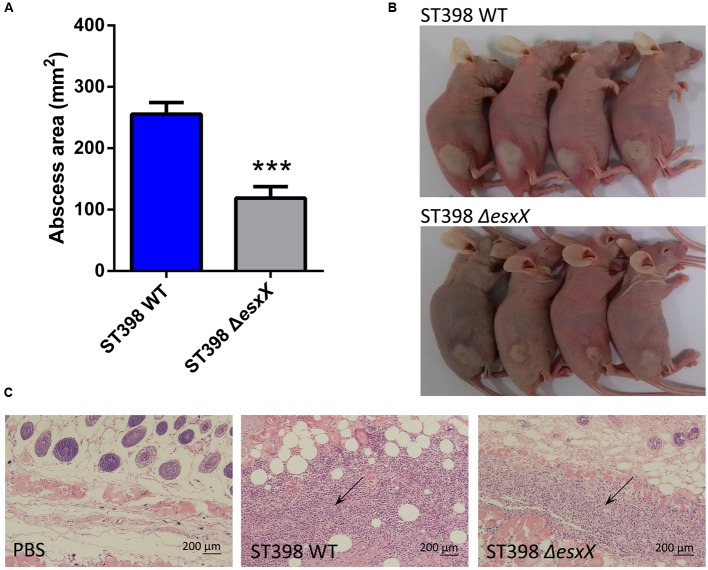
**Role of EsxX in CA-SA ST398 skin infection.** Mouse abscess model comparing the ST398 wild type isolate with an isogenic *esxX* deletion mutant. Control animals received only sterile PBS. **(A)** Abscess areas on day 2 after infection. ^∗∗∗^*p* < 0.001 (unpaired *t*-test). **(B)** Representative abscesses on day 2 after infection. **(C)** H&E staining of abscess skin tissue harvested on day 4. Note the considerable infiltration of inflammatory cells and damage of the subcutaneous structure in the wild type and *esxX* mutant samples; this infiltration is noticeably stronger in the wild type sample (arrows).

**FIGURE 6:**
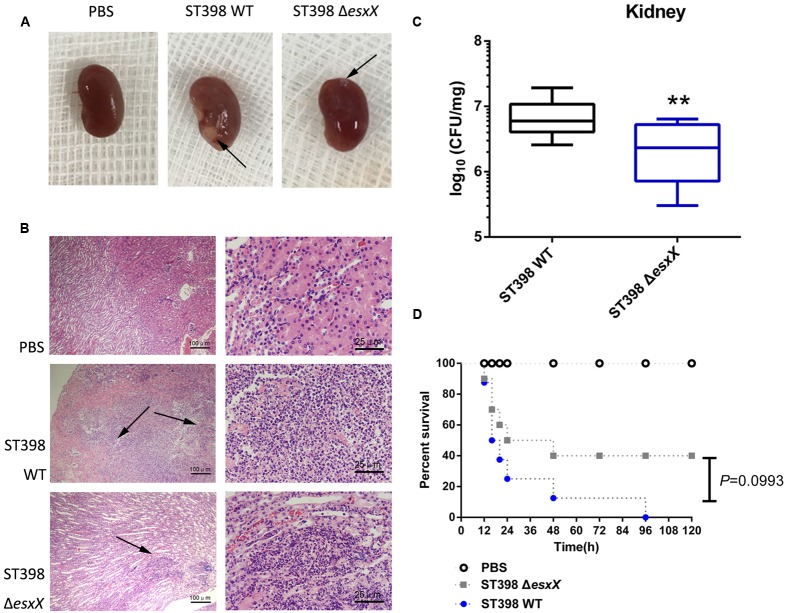
**Role of EsxX in CA-SA ST398 bacteremia.** Mouse bacteremia model comparing the ST398 wild type isolate with an isogenic *esxX* deletion mutant. Control animals received only sterile PBS. **(A–C)** Mice (*n* = 8 per group) were infected with 10^8^ CFU of *S. aureus* and euthanized 4 days after injection. **(A)** Representative macroscopic abscess formation in kidneys of mice infected with ST398 wild type and the *esxX* mutant strain (arrows). **(B)** Histological results of representative kidneys. Note pronounced infiltration of inflammatory cells and structural destruction in the wild type sample, while significantly less infiltration and damage was evident in the *esxX* mutant sample (arrows). **(C)** The number of CFUs in the kidneys was determined by plating samples on TSB agar. ^∗∗^*p* < 0.01 (unpaired *t*-tests). **(D)** Survival analysis of mice (*n* = 8 per group) injected with 10^9^ CFU of S. aureus or PBS. Survival curves were compared using a log-rank (Mantel-Cox) test, *P* = 0.0993.

*Staphylococcus aureus* causes a variety of infections, including superficial skin and soft tissues infections (SSTIs) and severe invasion infections such as bacteremia. After evaluating the impact of EsxX on the capacity of neutrophil lysis *in vitro*, we then explored its virulence potential *in vivo* using mouse skin abscess and bacteremia models. In the skin abscess model, the ST398 caused a significantly larger abscess area than the *esxX* mutant (**Figures 5A,B**), and histological examinations showed greater infiltration of inflammatory cells and skin tissue destruction in the mice infected with the wild type than those infected with the *esxX* mutant strain (**Figure 5C**). In a low-dose (10^8^ CFU) bacteremia model, the kidneys of mice infected with the ST398 wild type showed increased abscess formation (**Figure 6A**). Histological examination showed increased infiltration of inflammatory cells and tissue structure destruction in the kidneys of ST398 wild type-strain-infected mice compared with those *esxX* mutant-strain-infected mice (**Figure 6B**). Furthermore, the mice infected with the wild type strain showed notably higher CFU counts in the kidneys 4 days after infection than the mice infected with the *esxX* mutant strain (**Figure 6C**). In a high-dose (10^9^ CFU) bacteremia model, the mice infected with ST398 wild type all died within 96 h, while the mice infected with the *esxX* mutant survived for a longer time (**Figure 6D**); however, there was no statistical significance (*P* = 0.0993). The results of skin and blood infections together suggested that EsxX makes a significant contribution to the virulence of ST398.

## Discussion

In recent years, an ongoing surge of CA-SA infections have gained increasing world-wide consideration (DeLeo et al., 2010; Dantes et al., 2013). Compared with traditional HA-SA, CA-SA infects otherwise healthy individuals with no predisposing risk factors or conditions, indicating that CA-SA strains have enhanced virulence. USA300, the epidemic clone of CA-MRSA in the United States, has received much attention, but data regarding CA-SA in China is limited. The CA-SA lineage ST398, not related to contact with livestock, has grown to be a considerable problem in China, as described in our earlier work. Unlike USA300, whose epidemiological success has been linked to the acquisition of factors encoded on mobile genetic elements, such as the Panton-Valentine leukocidin (PVL), the arginine catabolic mobile element (ACME), and a comparatively small methicillin resistance element, SCCmec IV (or V) (Otto, 2010), CA-SA ST398 is showing increased expression of genome-encoded toxins such as PSMs and α-toxin and increased expression of the *agr* global virulence regulator. In addition to these common virulence factors, studies have revealed the important role of the ESAT-6 like secretion system (ESS) in USA300 (Burts et al., 2005; Korea et al., 2014). Recently, we have also described a previously unrecognized role of the ESS in the virulence of the CA-SA ST398 (Wang et al., 2016). In the present study, we compared the *ess* locus of ST398 with the published *S. aureus* sequence in the NCBI database and identified a novel protein, designated EsxX. We revealed this protein as a secreted factor and investigated its important role in the innate host defense and virulence of *S. aureus* ST398.

Initially, we found *esxX* located downstream of *esxB* in the *ess* locus of ST398, which is different from any other genes identified at similar locations of USA300. It is highly conserved in ST398, and there are no homologous proteins in other *S. aureus* strains. This finding might explain why there is no previous research about the function of EsxX, because the virulent CA-SA lineage ST398 was discovered only within the last few years. EsxA and EsxB, the two most studied ESS substrates, are both members of the WXG100 protein superfamily, which features a conserved “Trp-Xaa-Gly (WXG)” motif (Pallen, 2002). EsxX, which also belongs to the WXG protein family, has a unique LXG domain at the N-terminal. It is reported that the LXG domain is comparable to the WXG/ESAT-6 domains (Zhang et al., 2011), and they are both classified as signature N-terminal or pre-toxin-domains of the T7SS/ESS (Zhang et al., 2012), indicating that EsxX might be a substrate of ESS, similar to EsxA and EsxB. Furthermore, our data from the LC-MS/MS analysis and Western blots demonstrated that the secretion of EsxX is controlled by the ESS of ST398, and its subcellular localization also supported our hypothesis. In addition, EsxX has no effect on the secretion of EsxA and EsxB, suggesting that it might play a role independently. There are many secreted virulence factors that are *agr*-regulated, including EsxA and EsxB. Although EsxX could contribute to virulence, it is not under *agr* control, and this feature may be due to its strain specificity. Unlike EsxA and EsxB, which play roles in many *S. aureus* strains, EsxX is conserved in ST398, meaning that its role could be clone-dependent and does not require the engagement of the *agr* global virulence regulator.

Community-associated *S. aureus* ST398 showed enhanced virulence that promoted pathogenicity, and it was able to spread easily because of its poor biofilm formation. Although EsxX did not influence the growth, hemolysis, or biofilm formation of ST398, the observation of neutrophil killing and bacterial survival revealed that EsxX promoted neutrophil destruction and contributed to evasion of elimination by neutrophils. The microscopic analysis also showed the impact of EsxX on neutrophil lysis after phagocytosis. These findings suggested that EsxX plays an important role in resisting human innate defenses. Moreover, experimental murine skin and blood infection models showed EsxX to be a substantial contributor to both SSTIs and invasion infections. The significant influence of EsxX on virulence indicated that EsxX might have the potential to be a new virulence factor of ST398.

Our study demonstrated that EsxX is a novel secreted protein of ST398 ESS. EsxX is highly conserved and plays an important role in innate host defense and virulence in this epidemic CA-SA clone. However, the secretion mechanism of EsxX and how it interacts with host cells remain unclear. Pore-forming toxins contribute to bacterial pathogenicity by forming pores and/or disrupting host cell membranes (Los et al., 2013). Studies have shown that the ESAT-6 of *M. tuberculosis* has membrane-lytic and pore-forming activity (De Leon et al., 2012; Ma et al., 2015). However, no similar evidence regarding EsxA or EsxB in *S. aureus* has been reported thus far. It appears that the C-terminal domain (334–481) of EsxX shares structural homology with *E. coli* Colicin IA (PDB 1cii_A). Might it have pore-forming potential? Does the C-terminal domain of EsxX play a role in neutrophil lysis? Much work remains. Further investigation of this novel secreted protein might help us better understand the role of the ESS in ST398 and its contribution to the evolution of this virulent CA-SA lineage.

## Author Contributions

YD, YW, QL, QG, HL, HM, JQ, and MH conducted experiments. ML planned, supervised experiments, YD and ML wrote the paper.

## Conflict of Interest Statement

The authors declare that the research was conducted in the absence of any commercial or financial relationships that could be construed as a potential conflict of interest.

## Footnotes

1 http://www.mlst.net
