# Supplementary material for: A Novel ESAT-6 Secretion System-Secreted Protein EsxX of Community-Associated Staphylococcus aureus Lineage ST398 Contributes to Immune Evasion and Virulence
Source: Front Microbiol. 2017 May 5;8:819. doi: 10.3389/fmicb.2017.00819 (PMC5418362; doi:10.3389/fmicb.2017.00819)
Supplement: Supplementary file 1 [file Table_1.DOCX]

**Supplementary Table 1 Sequence alignment of the** ***esxX* gene in clinical isolates**

| **Isolation of ST398** | | | | |
| --- | --- | --- | --- | --- |
| **No. of ST398 isolates** | | ***esxX* gene sequence alignment** | | |
|  |  | **No./total** | **Percentage (%)** | **Sequence identity** |
| human | cattle | 66/76 | 86.8 | 100% |
| 61 | 15 | 10/76 | 13.2 | 99% |
