# Supplementary material for: A Novel ESAT-6 Secretion System-Secreted Protein EsxX of Community-Associated Staphylococcus aureus Lineage ST398 Contributes to Immune Evasion and Virulence
Source: Front Microbiol. 2017 May 5;8:819. doi: 10.3389/fmicb.2017.00819 (PMC5418362; doi:10.3389/fmicb.2017.00819)
Supplement: Supplementary file 2 [file Table_2.DOCX]

**Supplementary Table 2 BLAST results of *esxX* gene sequences in NCBI database**

| **Strain** | **Max score** | **Gaps** | **Sequence identity** | **ST type** |
| --- | --- | --- | --- | --- |
| ST398 | 2870 | 0 | 100% | ST398 |
| RIVM3897 | 2870 | 0 | 100% | ST398 |
| RIVM1607 | 2870 | 0 | 100% | ST398 |
| RIVM1295 | 2870 | 0 | 100% | ST398 |
| 08BA02176 | 2870 | 0 | 100% | ST398 |
| 71193 | 2863 | 1 | 99% | ST398 |
| 08-02119 | 2549 | 8 | 96% | ST582 |
| ST20130939 | 2549 | 8 | 96% | ST15 |
| ST20130938 | 2549 | 8 | 96% | ST15 |
| ST20130941 | 2549 | 8 | 96% | ST15 |
| ST20130940 | 2549 | 8 | 96% | ST15 |
